# Entrustment of the on-call senior medical resident role: implications for patient safety and collective care

**DOI:** 10.1186/s12909-017-0959-3

**Published:** 2017-07-14

**Authors:** Noureen Huda, Lisa Faden, Mark Goldszmidt

**Affiliations:** 1 Department of Medicine, Schulich School of Medicine and Dentistry, Western University, 1151 Richmond St, London, ON N6A 3K7 Canada; 2 Centre for Education Research and Innovation, Schulich School of Medicine and Dentistry, Western University, Health Sciences Addition, Suite 110, N6A 5C1, London, ON Canada; 3 University Hospital, Room B9-105, London, ON N6A 5A5 Canada

**Keywords:** Entrustable professional activities (EPAs), On-call supervision, Competency

## Abstract

**Background:**

The on-call responsibilities of a senior medicine resident (SMR) may include the admission transition of patient care on medical teaching teams (MTT), supervision of junior trainees, and ensuring patient safety. In many institutions, there is no standardised assessment of SMR competency prior to granting these on-call responsibilities in internal medicine. In order to fulfill competency based medical education requirements, training programs need to develop assessment approaches to make and defend such entrustment decisions.

The purpose of this study is to understand the clinical activities and outcomes of the on-call SMR role and provide training programs with a rigorous model for entrustment decisions for this role.

**Methods:**

This four phase study utilizes a constructivist grounded theory approach to collect and analyse the following data sets: case study, focus groups, literature synthesis of supervisory practices and return-of-findings focus groups.

The study was conducted in two Academic Health Sciences Centres in Ontario, Canada. The case study included ten attending physicians, 13 SMRs, 19 first year residents and 14 medical students. The focus groups included 19 SMRs. The later, return-of-findings focus groups included ten SMRs.

**Results:**

Five core on-call supervisory tasks (overseeing ongoing patient care, briefing, case review, documentation and preparing for handover) were identified, as well as a range of practices associated with these tasks. We also identified challenges that influenced the extent to which SMRs were able to effectively perform the core tasks. At times, these challenges led to omissions of the core tasks and potentially compromised patient safety and the admission transition of care.

**Conclusion:**

By identifying the core supervisory tasks and associated practices, we were able to identify the competencies for the on-call SMR role. Our findings can further be used by training programs for assessment and for making entrustment decisions.

## Background

Research on transitions in patient care has largely focused on discharge [1–4] and ignored the admission transition. In many academic health science centres (AHSC), the admission transition is particularly crucial as patients are admitted, from the emergency room, by the on-call senior medical resident (SMR) to medical teaching teams (MTTs) [5–8]. At present, our understanding of the on-call SMR activities that support patient safety and collective care on MTTs is limited. Consequently, this constrains our ability to assess the competencies required for the on-call SMR role, prior to making entrustment decisions.

The SMR role is a critical progression in internal medicine training. In many programs, internal medicine residents undertake the role of on-call SMR as of July 1st of their second year of training. Depending on the context, on-call SMRs may be expected to independently – with the attending available by phone – oversee all internal medicine care in the hospital overnight. This may also include supervising the junior trainees as they provide care for new emergency room consultations and previously admitted patients on the ward. The role also requires that SMRs ensure adequate handover to the MTT the next morning. However, recent studies exploring the adequacy of handover [9–13] and collective care [14] provided by MTT members point to the critical role of the admission transition. Specifically, Goldszmidt et al. observed that fragmented on-call documentation and incomplete information during handover impacted the ability of the MTT to provide safe patient care [14]. Consequently, the on-call SMR is central to the admission patient care transition.

In recognition of this pivotal role, strategies – largely involving making it safe for SMRs to seek support when on-call – have been proposed [15]. However, as Kennedy et al. [16] have demonstrated, the pressure on trainees to work independently may influence their decisions to seek support. Other potential identified barriers have included concerns over evaluations, a desire to be an effective team member, heavier workloads and limited availability of supervisors [17]. While junior trainees have all had opportunities to observe their senior residents enact components of the SMR role during their own on-call shifts, they may not have received any formal training to develop effective strategies for the role. Moreover, their assessment prior to this point would have focused on a very different set of competencies, ones that may not adequately predict performance on this one. Therefore, programs need to do more than develop strategies primarily centred on support seeking to ensure on-call SMR competence; programs must ensure SMR competency on-call.

**Fig. 1:**
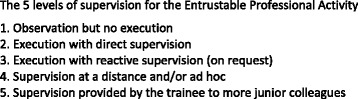
The 5 Levels of Supervision (Ten Cate, 2013)

Entrustment decisions for unsupervised activities, such as the on-call admission transition, are dependent on several factors. These include the trainee’s competency level, the complexity of the clinical activity, the availability of supervisors, and the level of supervision (Fig. 1) [18].

As the on-call SMR role is a level 4 activity, meaning that it must be performed without on-site supervision (Fig. 1),internal medicine trainee’s must demonstrate competency in performing the activities of this role prior to undertaking unsupervised on-call. However, our understanding of the on-call SMR activities is limited. This constrains our ability to make entrustment decisions for level 4 activities. The purpose of this study, therefore, was to explore SMR on-call clinical activities that support collective care and patient safety during the admission transition so as to better inform the assessment of competencies and entrustment decisions specific to the on-call role.

## Methods

A constructivist grounded theory approach [19, 20] was applied to this four phase study. Constructivist grounded theory applies a rigorous set of procedures to simultaneously collect and analyse multiple sources of data; to code and categorise data based on related themes; and to construct an emerging theory. The emerging theory further informs theoretical data sampling in subsequent phases of the study.

Ethics approval was obtained from the Western University (Reference: 16823E) and McMaster (Reference: 11–409) University Health Sciences Research Ethics Boards. Written consent was obtained from all participants for audio recording and for use of anonymized transcripts.

### Setting and participants

The settings were internal medicine wards in two AHSCs in Ontario, Canada. At each centre, there are three internal medicine MTTs that admit patients, from the emergency room, during the overnight on-call period. The on-call admission rate varies from 15 to 20 patients per night at each site. A majority of the patients present with multisystem disorders.

The on-call teams at both sites include an SMR (second or third year medical resident) and one junior trainee (senior medical student or first year resident) from each of the three teams. The SMRs on-call responsibilities include: providing patient care, supervising the junior trainees, reviewing the new admissions, and preparing the junior trainees for an independent case presentation to the attending physician the next morning.

In addition to supervising the admission transition of care, the on-call SMR also accepts transfers of care from the intensive care unit and, at times, provide acute care during in-hospital medical emergencies (code blue). Hence, in this setting, the on-call SMR role is more diverse than the MTT SMR.

### Data collection

Data collection took place in four phases designed to iteratively explore on-call SMR practices. In the first phase, as part of a larger study focused on collective care by MTTs [14], data was collected for 19 patient admissions. The data included de-identified clinical documentation; patient care orders; and audio-transcribed recordings of the on-call admission case review between the SMR and junior trainee and the morning case review between the attending physician and junior trainee. As the first phase did not include direct observation of the on-call SMR role, the data collection in phase two explored the outstanding questions from phase one and the emergent theory. Phase two consisted of focus groups with SMRs, who were not part of the first sample. The findings were then further elaborated in the third phase, which explored the existing literature to identify supervisory practices that support patient safety and collective care on MTTs. Specifically, phase one and three were crucial in identifying the essential practices that support the admission transition of patient care, as well as practices that were detrimental to the admission transition. The fourth phase included two return-of-findings focus groups with a new group of SMRs.

### Sample

The case study consisted of 19 cases from three MTTs. The cases were collected across two 8-week periods (summer and winter of 2010). The data collection periods were purposefully selected to sample a wider variation in trainee experience, weekday and weekend cases, on-call senior-junior pairings and attending physician expertise. The patient cases were a convenience sample (all cases where we had consent from the junior, SMR and attending physician). In total, there were ten attending physicians, 13 SMRs, 19 first year residents and 14 medical students.

Recruitment for the focus group commenced in 2012. Nineteen SMRs volunteered to participate in the data collection between February and November 2012. Following analysis of the focus group data, another recruitment letter was circulated for a return-of-findings focus group. This data was collected in December 2014. As the SMR cohort from phase two had graduated from internal medicine residency, this phase included 10 new SMRs.

The literature synthesis included 11 articles that addressed the influence of supervisory and communication practices on patient safety and collective care on MTTs (Table 1).

**Table 1:** On-Call Core Supervisory Tasks, Practices and Evidence from Literature Synthesis

1. Overseeing Ongoing Patient Care. Supervisors improve patient safety [32–36] by: identifying missed diagnoses, providing support during clinical uncertainty and ensuring trainees are involved in changes in the management plan [14].
Supervisory Practices
1. Complete full patient evaluation (SMR takes complete history and performs physical exam) 2. Conduct focused patient evaluation (SMR takes a brief history and may or may not examine patient)^a^ 3. Review prior clinical notes and investigations 4. Read around patient’s presentations as needed 5. Assign patient to a junior based on competency level 6. Support juniors with monitoring and managing their patients in the ER^a^ 7. Follow up results of investigations^a^ 8. Request support from peers during clinical uncertainty^a^
Identified Detrimental Practices
9. No patient evaluation completed (SMR only ensures that the patient is stable) 10. Independently monitor and manage admitted patients in the ER
2. Briefing Supervisors brief to set expectations for patient assessments [37] and to build collaborative plans [38].
Supervisory Practices
1. Guide juniors on key areas to focus assessment 2. Direct juniors on what to read before patient assessment 3. Guide juniors on information to obtain from prior clinic notes 4. Set expectations for the junior to complete their assessment & plan prior to case review
Identified Detrimental Practices 5. No briefing, assign patient only
3. Case Review Supervisor’s feedback on the organisation and content of a presentation supports accurate problem lists [39] and appropriate management plans [40, 41].
Supervisory Practices
1. Review case presentation in the conference room or by the bed-side^a^ 2. Provide feedback on organisation of case presentation & required contextual adjustments ^a^ 3. Demonstrate pertinent exam findings^a^ 4. Probe the junior around knowledge gaps around their assessment & plan^a^ 5. Coach and support the junior in developing their own problem list & plan^a^
Identified Detrimental Practices 6. Direct the junior on content of the problem list & plan (no coaching or probing of knowledge gaps)
4. Documentation Supervisors enhance the team’s ability to provide comprehensive patient care by ensuring that documentation is complete and consistent [42, 43].
Supervisory Practices
Admission Note 1. Explicitly direct the junior on where and what to amend in the admission note^a^ 2. Observe junior amending the admission note
Identified Detrimental Practices 3.Not asking the junior to amend the admission note
SMR Note 1. SMR note contains the full problem list and plan^a^ 2. SMR does not write a note but ensures that the admission note contains the full problem list & plan^a^
Identified Detrimental Practices 3. SMR note contains part of the problem list and plan 4. SMR does not write a note and does not ensure that amendments are made to the admission note
Patient Care Orders 1.Reconcile patient care orders with the full problem list and plan^a^
Identified Detrimental Practices 2.Orders are not reconciled with the full problem list and plan
5. Preparing for Handover Supervisors can ensure a safe handover by: prioritising issues for handover [10–12], flagging pending investigations [44], and acknowledging problems that could not be fully explored during on-call [10].
Supervisory Practices
1. Prioritise issues on problem list with junior^a^ 2. Highlight patient care issues that could not be addressed and need further follow-up by the team^a^ 3. Flag for junior which investigations are still pending^a^ 4. Inform the junior about the attending physician’s preferred case presentation style
Identified Detrimental Practices 5. No handover preparation

^a^Essential practice

### Data analysis

NVivo9™ qualitative data analysis software was used for data handling. The coding team included: N.H. (attending physician and medical educator), M.G. (attending physician and communication expert), and L.F. (research scientist). N.H. analysed a subset of the data to define the initial codes. M.G. and L.F. reviewed the codes to refine them further and to identify discrepancies. The refined codes were applied to a second subset of data. M.G. and N.H. continued to iteratively refine and reapply codes until the definitions were consistent and any new codes had been accounted for. The finalised codes were applied to a larger subset of data. These codes were categorised and compared within and across data sets for relationships that gave rise to emerging themes. The emerging themes and their relationships were reviewed by all three members of the coding team prior to analysing the remainder of the data set.

Rigour was supported by the following strategies: investigator triangulation of findings; triangulation of the three data sets; verification of outlier findings in the return-of-findings interviews; and documentation of analytical memos [21].

## Results

**Fig. 2:**
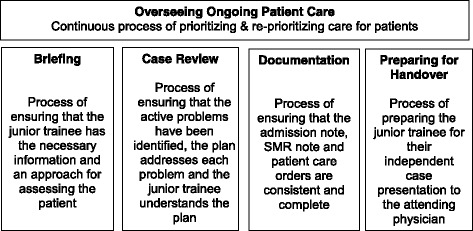
Core On-Call Supervisory Tasks

Five core on-call supervisory tasks (Fig. 2) that support collective care and patient safety were identified: overseeing ongoing patient care, briefing, case review, documentation and preparing for handover.

Overseeing ongoing patient care was a continuous task that applied to all the emergency room and MTT patients. Hence, this occurred concurrently with the other four tasks. The other tasks were specific to each new admission and occurred sequentially.

We also identified a range of practices associated with each of the core tasks (Table 1). Essential practices supported collective care and patient safety and were identified in phases one and three of data collection and analysis. In contrast, detrimental practices presented threats to patient safety and collective care. Phase two identified the variations in practices as well as contextual challenges that influenced how SMRs configured and used practices to achieve each task. Some SMRs described utilizing a diverse range of practices, others appeared to have more limited repertoires. A full range of practices was not necessarily the ideal and not all limited practice approaches were unsafe (Table 1).

In the following sections each supervisory task is reviewed. A description is provided of: the task, the essential and detrimental practices, and the challenges that led SMRs to deviate from their preferred practices.

### Overseeing ongoing patient care

**Fig. 3:**
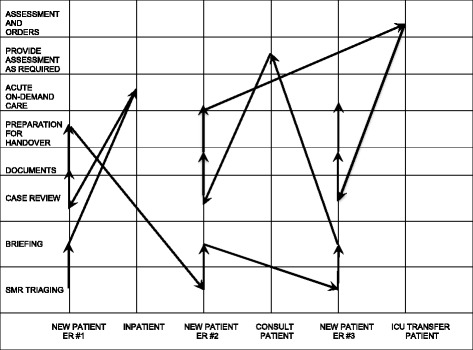
Visual representation of how SMRs sequence the on-call tasks

Overseeing ongoing patient care is a process of prioritizing and reprioritizing care for patients to maintain patient safety and efficiency. In the contexts studied, SMRs are responsible for overseeing the care of new patients in the emergency room as well as patients admitted to the MTTs. As a result, while the other core tasks are performed sequentially, Triaging overlaps with the other core tasks (Fig. 2). As SMRs also receive multiple patient referrals in a short span of time, often while they are in the midst of reviewing an existing referral, they may interrupt the sequence of tasks in order to triage efficiently. Figure 3 is a simplified visual representation of how an SMR might sequence a series of tasks on-call. At times, the junior trainees may also raise concerns regarding an already admitted patient. Accordingly, SMRs are constantly prioritizing and re-prioritizing which tasks need to be done and by whom.

As can be seen in section 1 of Table 1, four out of ten practices were found to be essential. The first was performing an independent evaluation of the patient. While an SMR could perform a full evaluation, it was only essential to perform a focused evaluation. The second essential practice was following up on investigations to ensure that urgent management issues (e.g. hyperkalemia) had been attended to prior to assigning the admission to a junior trainee. The third essential practice was including the junior trainees in ongoing patient care. The fourth essential practice involved collaborating with the emergency room physician around patient flow: “If I get 6 consults and I cannot look after everyone safely, the ER docs will say ‘don’t give X any patients until he is all caught up” (SMR21).

The SMRs described how patient context could change their preferred triaging practices. If the patient was stable with a straightforward presentation, then the SMRs could perform a limited evaluation: “Do I need to see this patient right now or do things sound straightforward and I am confident the junior can handle this?” (SMR1). Similarly, SMRs felt it was important to determine which junior trainee to assign each patient to: “If it was a low acuity patient, then maybe the medical student, but someone who is sick, goes to a resident” (SMR18).

The SMRs also described how on-call challenges could shift their preferred practices to ones where they were more or less personally involved. For instance, SMRs who described using the focused patient evaluation shifted towards a full evaluation if the patient’s presentation was unclear. Similarly, SMRs described performing a full evaluation, reviewing prior clinical notes, and reading around patients when they were still early in their senior year and felt inexperienced.

The two identified detrimental practices were: not performing a patient evaluation and independently managing patients without the knowledge of the junior trainees. The first practice raised concerns for patient safety, while a failure to involve junior trainees in the management of their patients impacted collective care on MTTs. As SMR22 recalled, “this morning the [post-call] med student had no idea about half the stuff that the senior had done [last night]. It was a disaster for us to review in the morning.”

### Briefing

Briefing is the process of ensuring that the junior trainee has the necessary information for assessing the patient. Section 2 of Table 1 lists the range of briefing practices. While no essential practices were identified, the four practices that may support collective care included: 1) guiding the junior trainee on key areas to focus the assessment; 2) relying on the junior trainee to obtain relevant information from prior clinical documents; 3) directing the junior to read around topics that can help with their assessment; and 4) setting expectations for their case presentation. Failure to enact briefing practices was identified as being potentially detrimental to collective care. The patients referred to the on-call team often have multiple comorbidities and inexperienced junior trainees may not recognise which medical issues are pertinent to the current assessment.

Challenges, such as the on-call work load, could shift the SMRs preferred briefing practices towards a more limited range. SMR14 reflected on these challenges from a recent on-call: “you are constantly getting paged, running to the floor, code blues and by the time you come [to the emergency room] everyone is waiting for you.”

### Case review

Case review is the process of reviewing the case to ensure that the active problems have been identified, that the plan addresses each problem or indicates which problems need to be addressed by the team in the morning, and that the junior trainee understands this plan. During case review, the junior gives a standardised oral case presentation and also reports information that the SMR may have specifically asked for during briefing.

Section 3 of Table 1 lists the range of practices. By comparing the on-call and morning case presentation transcripts from the first cycle of the study, five practices were identified as essential for supporting collective care: 1) reviewing the full case presentation in a conference room or by the bed-side; 2) providing feedback on how to organise and adjust case presentations based on patient context; 3) demonstrating physical exam findings; 4) coaching on formulating a complete problem list and; 5) supporting the junior trainees in developing the patient care plan (Table 1).

The SMRs described how on-call challenges influenced which case review practices they were able to use. Multiple practices were used when they were working with novice junior trainees. However, if the junior trainee was fatigued, the SMRs were sensitive to how much of the case presentation they were going to critique: “if you try and teach [around] too many issues past 4am, then it doesn’t stick” (SMR8).

**Fig. 4:**
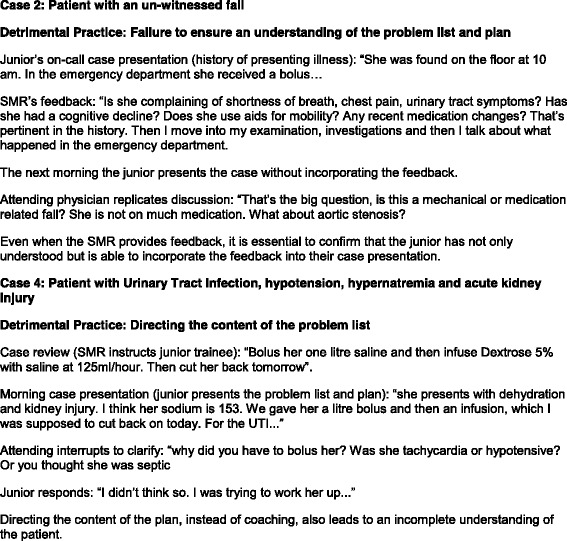
Examples of Detrimental Case Review Practices

Detrimental practices were identified as instances of clarifications and replicated discussions by the attending physician in the morning case presentation. These instances occurred around content that had been reviewed by the SMR but not presented by the junior, as well as deficits in the presentation that the SMR did not find time to critique. Figure 4 demonstrates case review practices that can be detrimental to collective care.

### Documentation

Documentation is the process of ensuring that the following admission documents are consistent and complete: the admission note by the junior trainee, the “senior note” by the SMR and the patient care orders. As the junior trainees write the admission note prior to case review, it is important to amend the documentation following case review discussions to avoid discrepancies between notes.

Section 4 of Table 1 lists the range of documentation practices. The essential practices included: 1) explicitly guiding the junior on how to amend their note; 2) ensuring that the problem list and plan in the two notes is consistent; 3) if there is no SMR note, ensuring that the admission note is complete; and 4) reconciling the patient care orders with the problem list.

SMRs elaborated on challenges that led to the variability in documentation practices. While they recognised the significance of amending the admission note, it was challenging to guide a sleep-deprived junior trainee: “they won’t start writing because they’re tired” (SMR17). The SMRs were unsure of the purpose of their note in supporting patient care. As a result, the senior note may or may not be written depending on the on-call workload. In contrast, the following challenges necessitated an SMR note: critical patient presentations, patients transferred to the ICU and inexperienced juniors.

Detrimental documentation practices were identified from phase one of the study as instances of clarifications and replicated discussions during the morning case presentation and by the presence of incomplete patient care orders. These practices included: 1) not expecting the junior to amend the admission note; 2) not writing an SMR note when the admission note was incomplete; and 3) not ensuring consistency between the problem list and patient care orders.

### Preparing for handover

Preparing for handover is the process of preparing the junior trainees for their independent case presentations to the attending physician. In our study context, the morning handover presentations take place separately with each of the junior trainees’ respective teams. As a result, the SMR is not available for all handover presentations.

The ranges of practices are listed in section 4 of Table 1. The essential practices included: 1) prioritizing issues on the problem list, 2) flagging issues that could not be addressed, and 3) reminding the junior to follow pending investigations. Another practice was discussing with the junior trainee the attending physician’s preferred case presentation style (full presentations versus problem list format). While this practice did not appear to be detrimental or essential for collective care, it served to improve the efficiency of morning handover as SMR17 explains, “We tailor things to how our attending wants them and try to get through the day in a quick manner”.

The SMRs described various challenges that influenced which practices they were able to use. If the junior trainee was inexperienced, the SMRs spent more time in preparing them for handover: “I coach them the first time I am with them. It helps guide the story so that the attending doesn’t have to go back and do it” (SMR11). At times, SMRs had to limit their practices due to the patient care load and the junior trainee’s level of engagement: “if it is really busy at 4:00 in the morning, the junior does not want to be coached on how to deliver the case” (SMR13).

The main detrimental practice was not preparing for handover. In our study context, most patients presented with multiple medical problems and not all problems could be addressed during on-call. Hence, it was important to ensure that the junior trainee was able to handover which problems needed further investigation by the MTTs.

## Discussion

Our study offers four contributions to the existing literature on patient safety [9–13] and collective care [14]: the identification of a set of core on-call supervisory tasks (Fig. 2); the understanding of the relationship between the tasks, collective care and patient safety; the identification of essential practices and; the identification of detrimental practices. Training programs in the process of implementing Competency Based Medical Education (CBME) [22, 23] can use our findings, contextualized to their setting, for training and assessing trainees prior to making entrustment decisions [24, 25].

Comparing our findings to the existing literature (Table 1), we were able to identify supporting empirical evidence for all of the core supervisory tasks except for briefing. While briefing has been proposed as an important supervisory task, we could not identify empirical evidence supporting its use in this context.

Our findings argue for the need to support “progressive independence” [26] for trainees so that granting progressive independence is balanced with formal training and assessment of competencies. Similar to the findings by Kennedy and colleagues [16], our study demonstrates that in the face of competing patient care demands, SMRs, at times, enact practices that could compromise the MTT’s ability to provide safe collective, patient care. This highlights the need for training programs to implement a more robust assessment method for entrustment decisions, in particular, the entrustment of SMR on-call responsibilities.

Critics caution that CBME may become reductionist if we do not appreciate the intricacies of clinical activities and how the clinical context influences physicians’ practice [27]. Our findings elaborate these concerns further. As demonstrated, the on-call SMR role involves not only demonstrating competency in the individual internal medicine Entrustable Professional Activities (EPA) [28] but also involves the balancing of competing on-call demands, and adjusting practices in the face of contextual challenges. As a result, advocates of EPAs [18, 29] propose that entrustment decisions should go beyond individual competencies and, instead, seek to understand the complexity of the clinical task; the desired outcomes of the task; and the level of supervision.

Ten Cate [23] proposes that training programs should consider clinical activities and their outcomes when making entrustment decisions [18]. Our findings demonstrate that the clinical activities of the on-call SMR role are the five core supervisory tasks. The key outcomes of the role are: supporting patient safety and collective care on the MTT. Proponents of CBME [30, 31] also suggest that training programs should consider the abilities required of trainees as they progress towards acquiring competence in clinical tasks. The range of essential practices explicitly demonstrates what abilities are required of trainees for the on-call SMR role. The practices also demonstrate how trainees can effectively modify their on-call activities in the face of organizational challenges. While our findings are transferable to many institutions where residents undertake SMR on-call, it is also important to recognize that our findings came from institutions with their own practices and contextual challenges. Each institution would have to explore how their context may shape these practices and tasks.

Our study has two main limitations. The lack of direct observation of the on-call SMR role prevented us from identifying all essential and detrimental practices. The focus of our inquiry was on the supervisory component of the on-call SMR role. Existing literature on supervisory practices suggests that teaching and supervision do not occur in isolation and are, in fact, interdependent [32]. Since we did not explore the teaching component of on-call supervision, it limits our ability to understand how SMRs configure their entire practice.

## Conclusion

In conclusion, our study is an important contribution towards understanding the core on-call tasks and practices that support patient safety and collective care on MTTs during the admission transition. Our findings demonstrate the key competencies that internal medicine trainees need to demonstrate prior to being entrusted with the on-call SMR role. The findings also have implications from a training and assessment standpoint. Future research should address how a CBME curriculum can integrate the SMR role as an EPA.

## Abbreviations

AHSC: Academic health science centres
CBME: Competency Based Medical Education
EPA: Entrustable Professional Activities
MTT: Medical teaching teams
SMR: Senior medical resident
